# Development and initial validation of a hospital stress questionnaire

**DOI:** 10.1080/21642850.2024.2396135

**Published:** 2024-08-28

**Authors:** Daniel M. Ford, Rebecca Lawton, Elizabeth Travis, Elizabeth A. Teale, Daryl B. O’Connor

**Affiliations:** a School of Psychology, Faculty of Medicine and Health, University of Leeds, Leeds, UK; b Quality and Safety Research Group, Bradford Institute for Health Research, Bradford Royal Infirmary, Bradford, UK; c Academic Unit for Aging and Stroke Research, University of Leeds, Leeds, UK

**Keywords:** Patients, stress, hospital, questionnaire, post-hospital syndrome

## Abstract

**Background:**

Hospitalisation can be a traumatic experience, where inpatients are exposed to an abundance of physical and psychological stressors. Evidence suggests that these hospital-related stressors negatively impact health: a phenomenon known as post-hospital syndrome. The current study aimed to identify hospital-related stressors, and to develop and provide initial validation for a new measure of in-hospital stress.

**Methods:**

Measure development occurred in three stages: (i) semi-structured interviews, (ii) item generation, and (iii) pilot testing. Twenty-one patients were interviewed regarding their recent hospital experiences, and a list of hospital-related stressors was produced. These stressors were compiled into a questionnaire and piloted on 200 recent inpatients to provide initial evidence of internal consistency and construct validity.

**Results:**

Stressors identified from the interviews captured all relevant questions from three previous hospital stress measures, plus 12 more. The most reported stressor was ‘poor sleep’. These hospital-related stressors were developed into 67 questions, forming the Hospital Stress Questionnaire (HSQ). The HSQ showed excellent internal consistency and construct validity, and correlated with feelings of vulnerability and being unprepared to go home.

**Conclusion:**

The HSQ is a promising self-report tool for measuring in-hospital stress. Future research ought to investigate its psychometric properties further in larger and more diverse samples. The measure has potential to be used to monitor patient risk of post-hospital syndrome.

## 1. Introduction

### 1.1. Background

Hospitals are widely considered to be a stressful environment, such that the experience has been described as ‘the trauma of hospitalisation’ (Detsky & Krumholz, 2014). In their paper, Detsky and Krumholz (2014) suggest that the traumatic nature of hospitalisation could be reduced by addressing a range of psychological (e.g. depersonalisation and uncertainty) and physiological (e.g. poor nourishment and rest) hospital-related stressors (see also Goldwater et al., 2018). These stressors are considered to be a significant factor in the aetiology of a phenomenon known as post-hospital syndrome (PHS): an acquired period of generalised vulnerability to major adverse events following hospitalisation (Krumholz, 2013).

This theorised relationship between hospital-related stressors and health has been bolstered by recent reviews highlighting the cumulative science linking psychological stress to negative health outcomes (O'Connor et al., 2021), and more specifically, in-hospital stress and patient outcomes (Ford et al., 2023). If a robust measure of hospital-related stress were developed and validated, it would facilitate evaluation of stress-reducing interventions of inpatients, inform policy decisions in hospitals, and could potentially be used as a predictor of patient outcomes.

### 1.2. Existing measures of hospital stress

Several measures of hospital stress have previously been developed, the first of which was the Hospital Stress Rating Scale (HSRS; Volicer, 1973, 1974; Volicer & Bohannon, 1975). The HSRS is a 49-item questionnaire, where each item details a stressful hospital-related event; items each have a pre-assigned rating of how stressful they are, and are presented from least (‘Having strangers sleep in the same room with you’) to most (‘Thinking you might lose your sight’) stressful. Participants respond by selecting which events they have experienced during their hospital stay, and the pre-assigned ratings of each item selected are summed to create a total score. This approach is potentially flawed when measuring subjective concepts such as stress: the preassigned rating of items does not allow for individual differences in perceived stress amongst patients. Additionally, some of the ratings are questionable, as higher ranking events (e.g. ‘Thinking you might have cancer’, rated 39.2) are likely to be more than just 2–3x as stressful as lower ranking events (e.g. ‘Having to eat at different times than you usually do’, rated 15.4). Although the measure was developed 50 years ago, many of the stressors included are still relevant for current inpatients. However, some questions are outdated or not relevant to the UK (e.g. health insurance), and the measure has not been sufficiently validated by contemporary standards (see Tsang et al., 2017).

Subsequent measures have been developed to identify and measure the stressors of specific groups of patients. The Intensive Care Unit Environmental Stressor Scale (ICUESS) is an adaptation of the HSRS for postoperative surgical ICU patients (Ballard, 1981), later extended upon and renamed the Environmental Stressor Questionnaire (ESQ; Cochran & Ganong, 1989). The Hospital Stress Index (HSI) is a list of 40 hospital-related experiences perceived as stressful, split into seven categories, designed specifically for elderly medical inpatients (Koenig et al., 1995). Participants respond to each item by selecting either ‘yes’ (scored as 1), ‘no’, or ‘unsure’ (both scores as 0) – however, this approach assumes that each event is equally stressful and, like the HSRS, does not accommodate for individual differences in perceived stress amongst patients. Moreover, the HSI has not been validated and lacks generalisability to other conditions as 89% of the patients in the sample informing the items were hospitalised with cardiovascular pathologies.

The most recent development sought to measure the hospital-related stress of elderly inpatients in an Iranian population (Hospitalisation-Related Stressors Questionnaire for Elderly Patients, HRSQ-EP; Musavi et al., 2016), later adapted and validated in Turkish (Yildirim & Işik, 2021). The importance of culturally sensitive health measures such as these has been emphasised (Rosa et al., 2010) in order to maintain the content validity of the instrument across different cultures (Beaton et al., 2002). This scale improves on previous measures as it utilises a 5-point Likert scale, allowing for individual differences in perceived stress. However, the development of the measure is poorly reported, which questions the reliability of the results; only seven patients were interviewed to inform the items, and no details are given regarding the interview participants, procedure, or results.

### 1.3. Aims of the current study

The aim of the current study was to identify hospital-related stressors experienced by inpatients, and to collate these findings with previous questionnaire items to develop a self-report questionnaire measuring in-hospital stress. This measure will be specific to the United Kingdom, generalisable across age groups and treatments (excluding paediatric, maternity, and psychiatric), and allow for individual differences in perceived stress. We then aim to provide initial validation for this novel questionnaire.

## 2. Method

Ethical approval was granted by the University of Leeds, School of Psychology Research Ethics Committee (development: PSYC-282; validation: PSYC-774), and informed consent was obtained from all participants. Development and initial validation of the questionnaire consisted of three stages – (i) qualitative interviews, (ii) item generation, and (iii) pilot testing to provide initial validation.

### 2.1. Stage 1: Qualitative interviews

#### 2.1.1. Participants

Twenty-one participants were recruited via social media platforms, word of mouth, snowball sampling, and Care Opinion (careopinion.org.uk). An invitation poster was uploaded to social media, detailing the inclusion criteria of the study, the most important of which was a recent hospital stay of 48 hours or more within the past 12 months. Each participant received a £20 gift voucher to compensate for their time. We sought to hear from all age groups, sexes, and ethnicities, and so purposive sampling was used to recruit from a diverse range of communities. Recruitment was terminated when a sufficiently diverse sample was achieved, and no new stressors were attained by interviewing further participants. A panel of lay leaders – independent representatives of patients and members of the public, with differing areas of expertise – were consulted to review the appropriateness of the recruitment material, and for assistance with contacting diverse and under-represented communities.

Participants were required to be over 18 years old and must have been an inpatient in a UK hospital for at least 48 consecutive hours in the 12 months prior to recruitment. This was to ensure ample time to experience a broad range of hospital-related stressors and is in keeping with previous work (Koenig et al., 1995). Participants that had been an inpatient for paediatric, maternity, or psychiatric care were excluded on the basis that the stressors associated with these settings would be vastly different to those associated with other conditions (e.g. Pichler-Stachl et al., 2016). Patients’ recollections of stressful hospital experiences have been shown to be reliable at 12 months following discharge (Löf et al., 2006), and so this length of time was chosen as the cut-off for recruitment. Two participants were inpatients more than 12 months prior to the interview but had materials to aid their memory (e.g. had taken notes during their hospital stay), and so were allowed to participate.

#### 2.1.2. Procedure

Using the framework set out by Kallio et al. (2016), semi-structured interviews were performed to identify the hospital-related stressors experienced by the participants during their hospital stays. An interview schedule was produced (see Appendix A), written using language suitable for a layperson and was rated as ‘easy to read’, according to the Flesch Reading Ease Score (FRES = 82.0). The schedule consisted of an introduction and 17 questions: 10 background questions and seven open-ended questions. Background questions were used to ensure a diverse sample of age, sex (Male, Female, Prefer not to say), education (No qualifications; GCSE/O-Level or vocational level 2 and equivalents; A-Level or vocational level 3 and equivalents; Undergraduate degree; Postgraduate degree; Prefer not to say), ethnicity (White; Mixed or Multiple ethnic groups; Asian or Asian British; Black, African, Caribbean, or Black British; Other ethnic groups; Prefer not to say), and reasons for hospitalisation. The open-ended questions began by asking participants to describe their experience of being an inpatient; this was intended to give the interviewer an understanding of the participant’s hospital stay, as well as an opportunity to follow up on any stressors that the participant identified in their account. Subsequent questions were designed to identify any further stressors that the participant omitted in their initial description. After the participant had spontaneously reported all of the stressors they recalled experiencing during their hospital stay, they were then prompted with known hospital-related stressors from previous academic literature – for example, Goldwater et al.’ (2018) stressors: sleep disruption, malnourishment, dehydration, mobility restriction, and pain – and asked if they had experienced any of these stressors during their stay. Finally, the participants were asked to rank the top three most stressful events that had been discussed within the interview. Interviews were audio recorded and later transcribed verbatim, with anonymisation, by the interviewer (researcher DF) for the purpose of data analysis.

#### 2.1.3. Data analysis

Interview transcripts were imported to NVivo 20.1.6 (QSR International Pty Ltd., 2020) and coded using quantitative content analysis (Neuendorf, 2017), with a positivist manifest approach. This approach is typically taken for a quantitative content analysis; it assumes objectivity, observability, and measurability of the data, and allows the researcher to generate observed frequencies of the codes (Kleinheksel et al., 2020). The purpose of the analysis was to identify and quantify the hospital-related stressors experienced by the participants during their hospital stays. To identify these stressors, all 21 transcripts were coded by researcher DF, 10 of which were then independently double-coded by researcher E. Travis. When quantifying the codes, frequencies of stressors were counted per participant, rather than per mention, to assess which stressors are most commonly experienced by inpatients. Stressors discussed in the interview that were not experienced as an inpatient were excluded from the analysis (e.g. parking, A&E, and health-related issues leading up to hospitalisation).

### 2.2. Stage 2: Item generation

Questionnaire items were generated in four phases. The first phase involved compiling the stressors identified in the interviews with those listed in previous hospital stress measures (HSRS; Volicer & Bohannon, 1975; HSI; Koenig et al., 1995; HRSQ-EP; Musavi et al., 2016). Second, the research team reviewed each stressor for cultural and contemporary relevance, removing any irrelevant or duplicate stressors, and the remaining stressors were posed as questionnaire items. Third, to assess the content validity of the questionnaire items, the measure was presented to the Yorkshire Quality and Safety Research (YQSR) Group – an inter-disciplinary team with extensive knowledge of patient safety and the embedding of health research into practice – who assessed the relevance of the measure and suggested additional items. Lastly, seven laypersons and three health professionals were invited to review the questionnaire to assess face validity. Two questions were asked: (i) ‘Did any of the questions not make sense to you? If so, which ones and why?’ and (ii) ‘Can you think of any stressful hospital experiences that were not on the list?’

### 2.3. Stage 3: Initial validation

#### 2.3.1. Participants

A sample of 200 participants was recruited from Prolific (www.prolific.co). To achieve a spread of ages, 100 participants were recruited using an age filter in the range of 18–49, and 100 were recruited filtering to select participants that were 50 years or older. In addition to this, participants were required to have stayed in a UK hospital as an inpatient, this stay must have been in the past 12 months for at least 24 hours, and not for paediatric, maternity, or psychiatric care. The inclusion criteria for length of hospital stay was shorter for this phase of the study as participants were no longer informing the items of the measure, and so were not required to have experienced as wide a range of stressors. For completing the survey, which took approximately 10 minutes, each participant was compensated with £1.50 via the Prolific payment system.

There are no absolute rules for the sample size required to validate a questionnaire (Osborne & Costello, 2004; Tsang et al., 2017); however, a widely-cited rule of thumb for psychometric theory is a subject to item ratio of 10:1 (Nunnally, 1967). Therefore, for a future companion study, researchers should aim to recruit 670 participants – this sample size is considered ‘very good’ (Comrey & Lee, 2013). While, for an initial validation, a sample size of 200 is recommended (Crocker & Algina, 1986; Frost et al., 2007) and so this number was adopted for the current study.

#### 2.3.2. Procedure

A survey was created using Qualtrics (www.qualtrics.com), and began with five screening questions (e.g. ‘Have you stayed in hospital in the UK?’), six demographic questions (e.g. age, sex, ethnicity, and education), and three questions relating to the post-hospital period: (i) ‘How prepared did you feel to go home when you left hospital?’, (ii) ‘How long after leaving hospital did it take you to get back to the usual activities you did before going into hospital? (e.g. driving, work, cooking, housework, leisure, etc.)’, and (iii) ‘In the six weeks after leaving hospital, how vulnerable did you feel? E.g. feeling weak, unsafe, or that your health might get worse.’ These three questions were designed by the research team to capture the effects of post-hospital syndrome, and were included to provide initial testing of the predictive validity of the questionnaire.

The above single item questions were followed by two questionnaires. First, the novel hospital stress questionnaire developed in stages 1 and 2, comprising of an introduction, 67 hospital-related stressors (rated 1 (not at all stressful) to 10 (extremely stressful) or ‘N/A’), an ‘Other’ option to include any additional stressors (rated 1–10 or ‘N/A’), an overall stress rating (rated 1–10), and an additional comments box. Three attention check questions (e.g. ‘Please select “7” to show you are paying attention’) were dispersed throughout the 67 items; participants failing more than one attention check were rejected, as per the Prolific Attention Check Policy. Second, the Perceived Stress Scale, 10-item version (PSS-10; Cohen et al., 1983), was included to assess convergent validity. Each of the 10 questions were reworded to begin with ‘While in hospital … ’ rather than ‘In the last month … ’ The PSS-10 was chosen as it is one of the most widely used measures of psychological stress, and has consistently been shown to be reliable and valid (Lee, 2012). The 10-item version was chosen over the 14-item version due to its shorter length, and chosen over the 4-item version due to its superior internal reliability (Cronbach’s alpha: 0.78 vs 0.60; Cohen, 1988a).

#### 2.3.3. Data analysis

Data were analysed using R Statistical Software (v4.3.1; R Core Team, 2023) (data and code can be accessed at: https://github.com/DMFord97/Initial-Validation). Descriptive statistics were used to analyse demographic data and responses to individual items. Cronbach’s alpha was employed to assess internal consistency, where a value of 0.7 or above indicates adequate consistency between items (Nunnally, 1967). To assess convergent and predictive validities, correlation coefficients were calculated, where *r* = 0.10, 0.30, and 0.50 were considered small, medium, and large, respectively (Cohen, 1988b).

## 3. Results

### 3.1. Stage 1: Qualitative interviews

#### 3.1.1. Descriptive statistics

Interviews ran from January to October 2022 and took place online (*n* = 19) or via telephone for those participants without access to the internet (*n* = 2). Each participant completed a one-to-one interview, lasting 32 minutes on average, ranging from 15 minutes to one hour. Participants were majority white and female, but overall the sample was diverse in age, sex, ethnicity, and education level (see Table 1). The median and mode lengths of stay were seven and five days, respectively, with stays ranging from two days to three months. On average, participants had been admitted to hospital six times in their life previous to the current stay, ranging from no previous stays to over 20. Participants had been hospitalised for surgical (*n* = 10) and medical (*n* = 11) treatments for a range of conditions: six patients were hospitalised for a gastroenterology condition, five for oncology, two for respiratory, two for COVID-19 related conditions, and one of each for cardiology, ear, nose, and throat, general surgery, nephrology, orthopaedics, and urology.

**Table 1.** Demographic data of semi-structured interview participants.

Demographic	Female	Male	Total
Age (years)			
18–24	1	1	2
25–34	3	1	4
35–44	2	3	5
45–64	4	1	5
65+	4	1	5
Ethnicity			
Asian / Asian British	2	2	4
Black / African / Caribbean / Black British	2	2	4
White	10	3	13
Education			
GCSE (or equivalent)	2	0	2
A-Level (or equivalent)	3	2	5
Undergraduate degree	5	2	7
Postgraduate degree	4	3	7
Total	14	7	21

#### 3.1.2. Quantitative content analysis

From the interview transcripts, 66 hospital-related stressors were identified. Table S1 (see Appendix B) records each of these stressors, the number of participants that reported experiencing them, and an illustrative quote. Of the 21 participants, 19 reported experiencing some level of sleep disruption. Other commonly reported stressors were ‘loss of control’, ‘pain’, and ‘noise’. Stressors such as ‘having to wear a hospital gown’, ‘being unfamiliar with the hospital rules’, and ‘missing small comforts’ were reported least frequently.

### 3.2. Stage 2: Item generation

#### 3.2.1. Item development

The hospital-related stressors identified from the interview transcripts were used to inform the items of the HSQ. Three codes were not developed into questions: ‘mental distress’ was considered to be a result of the stress, rather than a stressor in itself; ‘no visitor policy’ was specific to the COVID-19 pandemic; and ‘poor discharge’ was excluded as questionnaire respondents will be inpatients, and so will not have experienced the discharge process at the time of completion. The stressors ‘mobility restriction’ and ‘confined to bed or ward’ were combined and captured by one question: ‘Feeling like you could not leave your bed or ward’.

The above processing of the interview data generated 62 questionnaire items, which were then compared against previous hospital stress measures. Three measures were deemed appropriate for comparison (HSRS: Volicer & Bohannon, 1975; HIS: Koenig et al., 1995; HRSQ-EP: Musavi et al., 2016; Yildirim & Işik, 2021) as they were not designed for treatment – or setting-specific populations (e.g. ICUESS: Ballard, 1981). Table S2 (see Appendix C) illustrates the comparison process of the three existing measures with the quantitative content analysis (QCA) from the current study: the QCA identified 12 new stressors and all but four of the combined 111 questions from these three existing measures, two of which were added (‘Needing help going to the bathroom’ and ‘Worrying that your appearance might change (e.g. scars)’), and two of which were excluded as they related to the pre-hospital period (HSRS: ‘24. Being put in the hospital because of an accident’ and ‘34. Having a sudden hospitalization you weren’t planning to have’). Two questions were then recommended by the YQSR Group, informed by their own hospital-related expertise (‘Being transferred between wards or hospitals’ and ‘The hospital not meeting your individual needs (e.g. disability)’), and a final question was added by the research team, informed by their own stress-related expertise (‘Not being able to smoke, drink alcohol, or use other substances’). Table S2 shows each of the 67 items included in the HSQ, along with the source(s) informing that item.

#### 3.2.2. Questionnaire design

The total number of items in the questionnaire was 67, and concludes with two additional items: ‘Other (write in)’ and ‘Overall, how stressed did you feel during your hospital stay?’ For the complete HSQ, see Appendix D. A ten-point scale was chosen as it has been shown to be the most preferred scale length by respondents (Preston & Colman, 2000), and provides significantly higher reliability, validity, and discriminatory power than shorter scales (Preston & Colman, 2000). Laypersons reviewing the questionnaire requested that a ‘Not Applicable’ option be added to the scale, to distinguish between hospital-related stressors that were not experienced (rated ‘N/A’) and stressors that were experienced but not perceived as stressful (rated ‘1’). This request was met to improve usability; for the purpose of data analysis, ‘N/A’ responses will be coded as ‘1 (not at all stressful)’ as both answers indicate no stress experienced. No further stressors were suggested in the review process, and no items were highlighted as being difficult to understand. This was supported by calculating the Flesch Reading Ease Score of the introduction and all questionnaire items, which yielded an overall rating of ‘fairly easy to read’ (FRES = 75.7).

### 3.3. Stage 3: Initial validation

#### 3.3.1. Descriptive statistics

To assess the suitability of our sample, demographics of participants that completed the survey were compared against NHS hospital admission data from 2019–20 (NHS Digital, 2020), data was chosen from this year as figures from later years were affected by the COVID-19 pandemic. Sample demographics were representative in sex and four of the five ethnic groups, but had fewer participants in the ‘Black, Black British, Caribbean or African’ group. Education levels were varied in the current sample but were more educated than the general population. Table 2 shows the NHS figures for age and ethnicity, desired figures adjusted for a sample size of 200, and figures from the recruited sample (18–19 year olds were included in the 20–29 group). Ages within the recruited sample ranged from 18–84 (*M* = 45.83, *SD* = 15.74) but were younger overall compared to the NHS population, lacking in the >70 groups.

**Table 2.** Demographic data for NHS 2019–20 hospital admissions, with comparisons.

Demographic	NHS 2019–20 data		Desired figures		Actual figures	
	*n*	%	*n*	%	*n*	%
Age groups						
20–29	1,628,416	8.9	18	8.9	49	24.5
30–39	1,956,878	10.7	21	10.7	34	17.0
40–49	1,688,799	9.2	18	9.2	26	13.0
50–59	2,629,680	14.4	29	14.4	50	25.0
60–69	2,946,248	16.1	32	16.1	29	14.5
70–79	3,735,458	20.4	41	20.4	11	5.5
80–89	2,876,195	15.7	31	15.7	1	0.5
>90	816,206	4.5	9	4.5	0	0
Ethnicity						
Asian or Asian British	978,586	6.5	13	6.5	11	5.5
Black, Black British, Caribbean or African	467,431	3.1	6	3.1	1	0.5
Mixed or Multiple ethnic groups	215,140	1.4	3	1.4	7	3.5
White	13,006,263	86.5	173	86.5	177	88.5
Other ethnic group	375,423	2.5	5	2.5	4	2.0

#### 3.3.2. Initial psychometric validation

The HSQ total scores ranged from 69–567 out of 670 (*M* = 276.2, *SD* = 105.0). The top five highest and lowest rated stressors are reported in Table 3. None of the 67 items suffered from ceiling effects: for each item, percentages of respondents scoring the highest option (10) ranged from 0.5–16%. However, seven items had 50% or more of respondents scoring the lowest options (N/A or 1), indicating substantial floor effects, and these items could be considered for exclusion: these items were #59 (77%), #67 (70%), #66 (62.5%), #13 (60%), #48 (59.5), #30 (59%), and #65 (50%).

**Table 3.** Top five items with highest and lowest mean scores.

Questionnaire item	*M* (*SD*)
12. Feeling bored	6.40 (2.64)
19. Missing loved ones	6.39 (2.88)
1. Not sleeping well	6.36 (2.51)
4. Staying in a noisy room	6.16 (2.79)
3. Having pain or discomfort from your treatment	6.04 (2.42)
13. The staff making a mistake that caused you harm	2.42 (2.39)
48. The staff not asking for consent before treating you	2.19 (2.03)
66. The hospital not meeting your individual needs (e.g. disability)	2.10 (1.99)
67. Not being able to smoke, drink alcohol, or use other substances	2.08 (2.14)
59. Not being able to pray or do other religious activities	1.58 (1.38)

Cronbach’s alpha was calculated to assess the internal consistency of the 67 items in the measure, which was deemed as excellent (*α* = 0.97). The mean inter-item correlation was also of an acceptable standard (*r* = 0.34), suggesting that items are reasonably homogenous while also sufficiently unique (Piedmont, 2014). The corrected item-total correlation of two items indicated a weak association with the overall measure (*r* < 0.3), #59 (*r* = 0.29) and #67 (*r* = 0.20), and exclusion of these item should be considered (Nunnally, 1967). However, no items were removed from the measure at this stage, in order to accurately replicate the results in a full validation. The corrected item-total correlations of the other 67 items were acceptable (*r* = 0.32–0.76).

Construct validity was assessed via convergent validity, where the HSQ total score was correlated with the PSS-10 total score: a large, positive correlation was observed (*r* = 0.77, *p* < 0.001), indicating strong construct validity (Swank & Mullen, 2017). Initial predictive validity was assessed by correlating the HSQ with three single-item questions relating to the post-hospital period. The HSQ correlated moderately with the questions relating to feelings of vulnerability (*r* = 0.36, *p* < 0.001) and preparedness to go home (*r* = −0.28, *p* < 0.001), but did not correlate with the question regarding return to activities (*r* = −0.04, *p* = 0.56).

## 4. Discussion

The current study produced a comprehensive list of hospital-related stressors experienced by inpatients, this is evidenced by the fact that the QCA informing the items of the HSQ captured all of the in-hospital stressors included in previous (non-specific) hospital stress measures, plus 12 novel stressors. The resulting 67-item questionnaire has been deemed appropriate by academics, clinicians, and patients. The current measure is a self-report questionnaire, allows for individual differences in perceived saliency of stressors, and is generalisable across specialties (excluding paediatric, maternity, and psychiatric), hospital-settings, and age groups.

Initial validation of the measure returned promising results, which the authors aim to replicate on a larger scale. Convergent validity indicated that the measure is assessing a stress-related construct, as a strong, positive correlation was observed between the HSQ and the PSS-10. Cronbach’s alpha suggested that the measure is internally consistent, although some items may not be appropriate; a larger companion study should employ factor analysis to reduce the number of items and group the remaining items into subscales. Additionally, correlating the HSQ with questions relating to vulnerability and preparedness to go home suggested that the measure may be an appropriate predictive tool, but further work is needed to confirm this.

In future research, the measure has potential to be used to identify those patients most at risk of suffering the effects of post-hospital syndrome. The measure would also be an appropriate tool for measuring inpatient psychological stress in intervention studies relating to the in-hospital environment (e.g. Pati et al., 2016) and inpatient stress management (e.g. Chalageri et al., 2021; Tuncay & Sarman, 2024).

### 4.1. Limitations and future study

Although the novel questionnaire improves upon previous hospital stress measures, and shows promising initial psychometric properties, there were a number of limitations within the current study. First, the sample recruited to provide initial validation of the measure was lacking in participants aged 70 and above; a demographic that accounted for approximately 40% of NHS inpatients in 2019–20. Should a full validation be conducted, researchers ought to rectify this lack of representativeness of older populations, as a matter of priority. Similarly, the validation sample was recruited entirely via an online recruitment platform (Prolific), and so is potentially not representative of the general population – a full validation study should recruit through various means, including offline methods (e.g. community groups and religious settings). Further, although the HSQ offers a comprehensive list of hospital-related stressors, completing a 67-item questionnaire is a burdensome task, and so a shorter version will also be explored for future use in hospitals. Lastly, neither phase of the study invited current inpatients to participate, therefore, in order to effectively administer the current measure in hospitals, the authors recommend that it be trialled on a diverse sample of inpatients in a future study.

### 4.2. Conclusion

The current multi-phase study developed a measure of inpatient psychological stress: the Hospital Stress Questionnaire (HSQ). The measure was informed by patient interviews and previous similar questionnaires, resulting in a 67-item self-report tool, including 12 items not identified in previous related measures. The HSQ shows promising initial validation. Future research ought to investigate its psychometric properties further in larger and more diverse samples. Once validated, the HSQ has potential to be used in measuring stress-reducing interventions for inpatients, and monitoring patient risk of post-hospital syndrome.

## Institutional review board statement

✓ The study was conducted in accordance with the Declaration of Helsinki and was approved by an Institutional Review Board/Ethics committee. See details under Methods.
× The study received an exemption from an Institutional Review Board/Ethics committee; See details under Methods.

## Supplementary Material

Supplemental Material

## Data Availability

All data and R code are available at the first author’s GitHub (https://github.com/DMFord97/Initial-Validation).
