# Supplementary material for: Development and initial validation of a hospital stress questionnaire
Source: Health Psychol Behav Med. 2024 Aug 28;12(1):2396135. doi: 10.1080/21642850.2024.2396135 (PMC11363734; doi:10.1080/21642850.2024.2396135)
Supplement: Supplemental Material [file RHPB_A_2396135_SM0099.docx]

**Appendix A – Interview Schedule**

*Interview schedule*

**Introduction**

- Unscripted greeting (“Hello”, “My name is…”, “How are you?”, etc.).
- Read the following script:

“I’ll be the person interviewing you. I’d like to start by thanking you for taking part and telling you a bit about the reason for this interview.

Without going into too much detail, being in hospital can sometimes slow down a patient’s recovery because of the stress they feel. I’d like to find out exactly what it is about being in hospital that can make a person feel stressed. That means there are no right or wrong answers. I just want to hear how you felt when you were in hospital, and if anything made you feel stressed.

I’m now just going to repeat a few things that you should have seen on the forms you read earlier. The interview will be voice recorded – is that okay? Taking part is your choice, so you are free to leave at any point, and nothing bad will happen. You can also skip any questions that you don’t want to answer – just let me know and we can move onto the next question. You will also be anonymous, and so whatever you say in this interview, no one will know it was you. Do you have any questions?

If you’re happy with what I just said, we can start now. It won’t take any longer than an hour. Can I turn on the voice recorder now?”

**Background Information**

Q1. What is your age?

1. 18 – 24
2. 25 – 34
3. 35 – 44
4. 45 – 64
5. 65 +
6. Prefer not to say

Q2. What is your sex?

1. Male
2. Female
3. Prefer not to say

Q3. What is your highest level of education?

1. No qualifications
2. GCSE/O Level (or vocational level 2 and equivalents)
3. A Level (or vocational level 3 and equivalents)
4. Undergraduate degree
5. Postgraduate degree
6. Prefer not to say

Q4. What is your ethnicity?

1. White
2. Mixed or Multiple ethnic groups
3. Asian or Asian British
4. Black, African, Caribbean, or Black British
5. Other ethnic groups
6. Prefer not to say

**Personal stress**

*Question:*

1. “What would normally make you feel stressed? I’m not talking about the hospital yet, just your day-to-day life.”

*Prompts:*

“What kind of things do you worry about?”

“Can you tell me about a time in the past where you’ve felt stressed?”

**Hospital context (quick questions)**

*Questions:*

1. “What was the reason for your most recent hospital stay?” AND WHEN?
2. “Did you have surgery?”
3. “How long were you in hospital?”
4. “Was it your first time in hospital?” [if “yes”, skip to Q12]
5. “How many times in your life have you been a patient in hospital?”
6. “When was the last time you were a patient in hospital *before* the time we mentioned earlier?”

**Hospital experience**

*Questions:*

1. “Could you quickly walk me through a typical day for you while you were in hospital?”
2. “Did you ever have any visitors?”

*Prompts:*

“Let’s start with the morning, when you would wake up.”

“What was your ward like? Were there lots of other patients?”

“Who were your visitors? How often would they visit?”

*Note for interviewer:*

Note down any stressors mentioned; elaborate on each of them once P has finished walking through their typical day.

**Potential hospital improvements**

*Questions:*

1. “What do you think could have been done better by the hospital ward you were in?”
2. “What do you think is already being done to improve the hospital ward?”

*Prompt:*

“You mentioned you didn’t like [stressor]. How do you think that could be improved?”

“Did you notice anything that you thought was being done well?”

**Known stressors**

*Main question:*

1. “I have a short list of things that other people have found stressful while they were in hospital: I’m going to read them one at a time and we can talk about each one. The first one is… [Question]”

List of known stressors (from previous research):

Note: only list stressors that have not already been adequately discussed.

| Stressor | Question | Prompt(s) |
| --- | --- | --- |
| Sleep disruption  (Stewart & Arora, 2018) | “Did you sleep well?” | “Did you get woken up a lot?”  “Was the bed comfortable?”  “Did you have any nightmares?” |
| Hydration (Oates & Price, 2017) | “Did you drink enough?” | “Did you ever feel dehydrated?” |
| Malnutrition (Franklin et al., 2011) | “Did you have a healthy diet?” | “Were you tube-fed?”  “What kind of food did you eat?” |
| Mobility restrictions (Smart et al., 2018) | “Did you feel like you could move around enough?” | “Did you get to walk around much?” |
| Physical pain (Gregory & McGowan, 2016) | “Were you in any pain from anything other than [reason for hospitalisation]?” | “Did you need to have many injections?” |
| Mental distress (Aass et al., 1997) | “How was your mood?” | “Did you feel down a lot?”  “Did you often feel on-edge? |
| Loss of control  (Taylor, 1979) | “Did you feel like you were no longer in control?” | “Did you feel helpless?” |

**Stressor saliency**

*Question:*

1. “Of all the things that made you feel stressed in hospital, what would you say are the worst three? Listing the worst one first.”

*Prompt:*

List all stressors the participant admitted to experiencing and ask them to pick the worst three.

**Final**

“That is all of the questions done – is there anything else important that you would like to say before we end the interview? Anything that you think we may have missed?”

Thank participant again for their time and terminate the interview.

**Appendix B – Example quotes of stressors**

| **Table S1**  *Hospital-related stressors identified: frequencies and examples* | | |
| --- | --- | --- |
| Hospital-related stressor | *n* | Example quote |
| Poor sleep | 19 | ‘No, because it was just very busy still. And like I could hear stuff going on. It was very bright and they just had to keep coming to like switch drips or like change stuff so like no it was very disturbed sleep.’ [P#19] |
| Loss of control | 15 | ‘I thought yeah, you go in, you want to feel comfortable, you're out of your own surroundings, everything is out of your control.’ [P#06] |
| Pain | 15 | ‘Muscle tension, aches and pains. I have that anyway, but it was worse than ever.’ [P#01] |
| Noise | 15 | ‘Well, people yelling, uh, people shouting, bed squeaking, wheels on the beds moving around, other instruments getting moved around, doctors talking… The lighting had a flickering sound where I was.’ [P#15] |
| Staff-patient communication | 14 | ‘Yeah, yeah, it was uh, they were male nurses. They were OK. They were doing their duty but not very verbal. Uh, they were just handing over the medicine, and were checking the body temperature and the blood and that's it.’ [P#18] |
| Staff too busy | 14 | ‘If you’re coming out of the shower it is like they won't have time to talk to you, just like... put your clothes on, let's sort you out or something. I need more time and you just want them to talk to you, but they just don’t have time for anything.’ [P#01] |
| Staff not caring or friendly | 13 | ‘It was like they're not listening and being sharp, and that kind of thing was going on.’ [P#21] |
| Waiting | 13 | ‘So I was trying to deal with that, and then there's this eternal wait as a patient, you don't know when the doctors are going to come.’ [P#08] |
| Food unsuitable | 12 | ‘So come lunchtime, very often the choice of food that I'd make wasn't there. There was no choice of a light or a convalescent diet. Or food that was easy to eat, especially being nauseated, there was generally things with chips or potatoes, which I don't eat too much of, and I certainly don’t eat sliced white bread. So it was very difficult to get a healthy diet. And of course I wasn't having a visitor that could bring me anything in, and there wasn't a shop trolley or anything.’ [P#08] |
| Mobility restriction | 12 | ‘No, no because uh, I was totally under, my pulse was under glucose and other syringes were attached to me, so I was totally, for 24 hours, I was bedridden.’ [P#18] |
| Not knowing | 9 | ‘But the waiting time, just the level of like just being left around waiting, not knowing what's going to happen and feeling like, you know, this is just getting worse and worse. Can we do something?’ [P#10] |
| Boredom | 9 | ‘And there's no form of entertainment for patients that would help them get relief of their stress, maybe something such as a television that could bring a bit of excitement to the patients.’ [P#14] |
| Mental distress | 9 | ‘I felt scared, anxious. I thought am I actually ever gonna leave this place?’ [P#15] |
| Adverse effects | 8 | ‘You know, the medication is kind of, you know, it cures certain things, but it creates other problems and actually managing that is probably part of the challenge for me.’ [P#10] |
| Fear health would deteriorate | 8 | ‘But I mean, yeah, that was the most stressful thing really, was just being worried for myself and my health, you know? Like, I suppose I didn't think I was gonna die, I felt reasonably confident I was gonna pull through this. But it's sort of the nagging thought in the back of my head, you know, given the death rate of what was going on. The obvious worry is, you know, how badly am I ill, you know?’ [P#16] |
| Staff not listening | 7 | ‘I just feel like they should listen to the patient, and then they should try to understand what the patient is telling them instead of suggesting.’ [P#11] |
| Disturbed by other patients | 7 | ‘Everything that she wanted. Demands, and she was screaming and, you know, making a lot of noise.’ [P#20] |
| Loneliness | 7 | ‘And then if I’m in the hospital all alone, without any support from maybe friends and family, that could also make it stress for me. Because at some point, maybe here I will have to take care of myself all alone.’ [P#11] |
| Missing loved ones | 7 | ‘I would say definitely the kind of, yeah, the experience of being by myself and not being able to speak to people, it was a relief when, you know, my mum phoned, or I was able to see [wife] when she came in for that minute.’ [P#10] |
| Staff made a mistake | 6 | ‘When the consultant came to see me, and I'd been trying to tell everybody that my arm was blue and painful. And several times he tried to tell me that it was just a muscle ache. And I said, “Why would my arm be purple for a muscle ache?” and finally he sent me for a scan, and then I was waiting in the ultrasound department and a doctor called me and he said, “Oh, you're the person that's here for the reassurance scan. Which arm is it?” I said “It's the one that's purple”, and he said, “Oh God”. He did a scan and I had a clot from below my elbow up to my neck, in the vein.’ [P#08] |
| Staff rude or unprofessional | 6 | ‘I said, “Excuse me…” and I said politely, I don't want to be upsetting anyone, “Excuse me, this isn’t hot, can I have a hot cup of tea, please?” She said, “It is hot. I just warmed the kettle up now.” I said, “It's cold”, she said, “Oh, you want to touch it?”’ [P#21] |
| Staff not responsive to buzzer or call | 6 | ‘And I called out to the staff to you know, “Can you come and, you know, aspirate me and get rid of this bile”, nobody came. And so, what I did was use my mobile phone to phone the hospital switchboard, get put through to the ward, to tell whoever answered the phone, the person in bed, blah, blah, requires your assistance as soon as possible, because he is basically drowning in his own bile, and that was the only time that anybody came to help me. Because they ignored the buzzer. They ignored me calling out.’ [P#17] |
| Disturbed by observations | 6 | ‘I think being woken up in the middle of the night – now, I understand they have to do their observations, but – when you're sleeping and being woken up every couple of hours, that can be quite stressful too.’ [P#05] |
| Relying on others | 6 | ‘I think that's something I worry about, because I have been admitted when I haven't been able to get out of bed, when I've had to rely on somebody getting me out of bed, and you suddenly realise, gosh, if I can't get out and walk, and get myself a drink off my side, and things like that. Then how do I do that without pressing a buzzer and getting an irate nurse?’ [P#06] |
| Confined to bed or ward | 6 | ‘The rest of the time I was basically, uhm, you know, just hold up in my bed or I had an arm chair next to my bed I could sit in. So I was just sat around.’ [P#16] |
| Feeling unsafe | 5 | ‘You are laid in bed, vulnerable surrounded by these people around you. Yeah, and it's really, actually not acceptable for a woman. You shouldn't be in here in your strange clothing in front of strange man.’ [P#21] |
| Concern for other patients wellbeing | 5 | ‘So, the doctor and the nurse came in the middle of the night and they're talking to her. The lady is not even on the planet, she's not connecting – she’s having a schizophrenic episode. I said “Please ring her family”, “Oh no, we haven't got her signed consent”, I said “She doesn't know what planet she's on! Ring her family. Get someone to speak to her, or ask them whatever…” you know, “We can't do that”. I said, “What about our protection? [Other patient’s name] here, who's had nearly having a heart attack, I've had an episode, what about our welfare? And hers?”’ [P#21] |
| Hospital disorganised | 5 | ‘At discharge, they lost my narcotics and said that I hadn't brought them in. And so I don't know where they went. It was always very difficult to get them regularly. My drugs and vitamins for my heart and my immuno-suppressed condition, it was very hard to get those on time and I did have some problems because I wasn't getting the drugs regularly.’ [P#08] |
| Sharing a room with other patients | 5 | ‘The washroom and bathrooms, there are really only one or two so it was really a big, uh, you can say obstacle, because I have other Gastro disease also so it takes me a lot of time for, uh, urinary and motion purpose. But due to other patients that also need to use the same washrooms, so I was really in discomfort in that way.’ [P#18] |
| Staff-staff communication | 5 | ‘And then when consultants come to talk to you, if it's not your consultant, they tend to keep asking you to go over everything, and that's stressful 'cause you think “Well, I've told somebody all of this, you know, the day before” or they'll sort of say “Oh well, can you just talk me through it? Can you just say to me about it? So how far have we come?” and you’re like, can you not read notes?’ [P#06] |
| Access to own medication | 5 | ‘I have some medication like my order narcotic pain relief which is given 12-hourly and it's helpful. But if I'm a patient, I just do what I need to do here in hospital, because all of my tablets were taken away, and I said “Because I'm alert, I believe I can have my pills, to manage my pills myself and inform you what I've taken”, and they said, no, that wasn't possible, so they took everything away.’ [P#08] |
| Hearing or seeing emergencies | 5 | ‘I remember when I was in the high dependency unit, I actually was begging them to get me out of there because it was so stressful to hear everything going on, like it was horrible and I was absolutely crying my eyes out’ [P#09] |
| Homesick | 5 | ‘At some point I was already getting tired of staying in the hospital. I was getting there because I just needed to go home, and maybe I was just tired of being indoors, staying in one room for days. And I was feeling like my life was at a standstill because I didn't go to work. I didn't go out to do things I would have done if I were at home.’ [P#13] |
| Hydration | 5 | ‘I spent a lot of time dehydrated at first when I was very ill. It was very difficult to get anybody to bring in a fresh jug of water.’ [P#08] |
| Overcrowding | 5 | ‘I was dropped off by my husband and I was given directions to I don't know what you would call it, but it was like a ward, but it wasn't. It was just like tiny little cubicles, where I was just given blood pressure, pulse, just talked through what the operation was about. But I felt it was almost like a cattle market. There was so many people there for operation. And it just felt so uncomfortable.’ [P#05] |
| Overhearing staff conversations | 5 | ‘And then I remember later on in the night I heard, it was her and one of the other night nurses, two of them seated, then one of them was like “when I had COVID I was lying at home out in the garden in the sun and talking to all… Oh, and I had a brilliant time off blah-blah.” And I was just thinking, you know, just literally you’re in a hospital currently in a respiratory ward looking at people, every single person in that ward was COVID positive.’ [P#02] |
| Cancellation or delay | 5 | ‘They only mentioned to me about surgery when it was going to happen, but obviously it was delayed.’ [P#15] |
| Not involved in treatment plan | 4 | ‘Just because I guess like they decided the treatment obviously, because they're the experts, but I feel like it was decided without like even telling me what was going on. So like it was all their decision and it just like they just came and it just happened. So I feel like treatment was just given to me and I didn't have really any say.’ [P#19] |
| Equipment lacking | 4 | ‘And there wasn't a proper shower chair in there, there was just a kind of like a plastic chair that visitors sit on. So it wasn't really stable, and that was in a wet room so it would get incredibly wet all the way up to the toilet pedestal. So walking across the floor was dangerous.’ [P#08] |
| Bloods taken | 4 | ‘And then she tried to get the arterial blood out of me, and she went in to one wrist, didn't get it, went into the other wrist, didn’t get it. Went back to the other one, got it, well got a bit and then she says “Well, I’ll have to try again because it was a mixed sample” and then she went into my other wrist and have been for the fourth time, at this stage I was crying with pain. I was very distressed, and she gave up.’ [P#02] |
| Disturbed by symptoms | 4 | ‘Like a typical day, well, I felt so sick, I felt so ill that I don't really know it's a little bit of a blur.’ [P#19] |
| No visitor policy | 4 | ‘I'm happy in my own company, so I couldn't have cared less if there wasn't any visitors allowed, but actually that's not true because I think I would have been a lot less stressed if I had been allowed visitors. Because, for example, see that breakdown I had because I thought I had an infection, that was because I was on my own, allowing those thoughts to fester and fester. Whereas if I'd had my family visiting, I'd have been able to talk about that with them and they’d have said, “Oh don't be silly, you're fine”, you know, or reassured me.’ [P#09] |
| Temperature | 4 | ‘I think the temperature for me was really warm. They’re thinking about everybody as a whole. But yeah, it's something I mentioned, but just they said that there's nothing they can do about it because it's there for everybody.’ [P#01] |
| Unfamiliar environment | 4 | ‘I wasn't even told where I could go and have a shower. I had to find my own way. And these are things that I think are very important. You need to be able to find your way about the place, and as I said to you, I always find it difficult when I'm in a strange place. And obviously it was a new hospital. It was first time I'd been there.’ [P#05] |
| Lack of cleanliness | 3 | ‘And one of them left a bloodied dressing on my bedside table. Well, that dressing stayed there for I think it was about seven or eight hours. OK, and it was an obvious bloody dressing and I'd been visited by various people trying to take blood out of me. Nobody cleared it up. Eventually, a nurse came along to do something, I can't remember what, and her hand touched the bloody dressing, which she flicked onto the floor.’ [P#17] |
| Worrying about loved ones | 3 | ‘I’d have a video call or phone call with my husband, trying not to upsetting him. He's 76, he’s older than me and has his own health care problems, and so I was trying to keep him as informed as I could… So my mood, I tried to keep myself cheerful. But I was anxious about my husband.’ [P#08] |
| Having to follow hospital schedule | 3 | ‘And like many people have routine, I don't want a cup of tea straight after my lunch, but that's when they give you a cup of tea, and you don't feel you can press your buzzer to ask them “Can I have a cup of tea now?”’ [P#06] |
| Lights | 3 | ‘They've always got electric lights on. Now, I don't have a problem with electric lights, I really don't, but when it's nice weather and what have you and they keep switching, you know, the lights are on full, you can't go anywhere where it's just natural light.’ [P#06] |
| Drips or tubes in body | 3 | ‘The main thing was the drip that was being put in my body. I could take about two to three drips a day, and that was really disturbing for me.’ [P#14] |
| Reminded of loved ones who passed away in hospital | 3 | ‘So then they transferred me over to [hospital name], which wasn't very fun because that's where my dad passed away.’ [P#15] |
| Staff did not ask for consent | 3 | ‘I suppose, one big thing I say is not being told by the staff why you're getting something done or explain to you what's actually happening. I find that the nursing staff tend to do that. Even in the emergency department, I remember having a swab shoved up my nose, like I already had my positive test. But I remember them coming at me with it, this girl, she was so rough. So, so rough, and she never told me she was going to do that or, you know, not even saying “Can I?” she didn't ask permission to even do that, just came at me with this thing and shoved it up my nose.’ [P#02] |
| Poor discharge | 2 | ‘And the other factor is your discharge. That's really stressful for a lot of people. I know it is for me because usually they're giving you new meds, and you get like taken from your bed space, because they need the bed space ASAP, to go and sit in a room and then you get told “Can you just go and sit outside the ward?” and you can be… the last time I was in it took them four hours’ [P#06] |
| Cannot do normal activities | 2 | ‘Like my daily job. I'm actually a fashion designer so I love to do my work, but I wasn't able to do that while in the hospital.’ [P#11] |
| Fear of hospital acquired infections | 2 | ‘It was a sort of medical geriatric ward, there were people with infections and all sorts of things outside of the room for me. Uhm, which used to freak me out a little bit because they'd come in without gowns on or gloves, and so that worried me that I would get cross-contaminated, and I did get cross-contamination.’ [P#08] |
| Lack of privacy | 2 | ‘And this nurse came and I said “I need to go to the loo”, she said “We’ll bring you a bed pan”. I said “No. I need to go to the loo because of…” – this is where part of the nightmare starts – it’s a mixed ward, men on both sides.’ [P#21] |
| Dehumanisation | 2 | ‘I think, a lot of the time when you're sent to hospital, you're just, you're just a figure. That's what you are, or you’re a number. You know, I hate this aspect, I was bed number one, and … it's offensive to just label someone as, you know, bed number one. Number one, you know, that’s not what you are. You’re a person. I think in health care as well, a lot of the times, you know, people are just looking at paperwork and looking at, you know, the numbers, and not actually treating the person.’ [P#02] |
| Financial stress | 2 | ‘We had a lot of problems in the family as well, financially. So if he [husband] took time off work, he’d lose his job.’ [P#01] |
| Life on hold | 2 | ‘On the day that I had my surgery, I became a great grandmother, so there was some, you know, it was a really interesting kind of time for my family as well, because obviously, um, that was quite hard that I was missing out on that’ [P#03] |
| Poor/no Wi-Fi or phone signal | 2 | ‘I think for a lot of people the most important thing is connection with the outside world, and I found that wherever I stayed, I had my operation in a hospital near Nottingham, uhm, I could not communicate with my family because there's no signal, in every hospital I've been in I haven't been able to use my phone.’ [P#03] |
| Uncertain of diagnosis | 2 | ‘And having to do describe what's going on with you to a doctor, it's not always easy 'cause you're not sure how to describe it sometimes.’ [P#07] |
| Worrying post-hospital | 2 | ‘It was, then, I was thinking about: how would I get home? What’s going to happen? Would I get any help and support there?’ [P#01] |
| Missing small comforts | 1 | ‘There’s just basics, just tea and stuff like that. That's all you need.’ [P#21] |
| Not able to do religious activities | 1 | ‘So yeah, I follow Islam, strong Muslim. I like to get my five prayers done but I obviously couldn’t really do that due to the pain in my leg at the time.’ [P#15] |
| Staff attending to other patients more than you | 1 | ‘I think the doctor should like really work more on attending to the black people. They should attend more like equally to the white people, 'cause they mostly attend to the white people than the black people. So, I didn't really like it.’ [P#12] |
| Unfamiliar with hospital rules | 1 | ‘I think, uh, any personal assistant, or, uh, I think was missing. Who can guides where we have to go from? Who you have to consult and what would the procedure if you are under NHS or you are doing private?’ [P#18] |
| Wearing a hospital gown | 1 | ‘You are laid in bed, vulnerable surrounded by these people around you. Yeah, and it's really, actually not acceptable for a woman. You shouldn't be in here in your strange clothing in front of strange men.’ [P#21] |

**Appendix C – Sources informing HSQ items**

| **Table S2**  *Sources informing each of the 67 questionnaire items of the HSQ* | |
| --- | --- |
| Questionnaire item | Source(s) |
| 1. Not sleeping well | - QCA: ‘Poor sleep’ - HSRS: ‘6. Being awakened in the night by the nurse’ - HSI: ‘1.3. Sleep disturbed by frequent tests, blood draws, medications’ - HRSQ-EP: ‘17. Disruption of routine and normal habits and behaviors such as sleep and resting, activity, and diet’ |
| 2. Feeling helpless or not in control | - QCA: ‘Loss of control’ - HSI: ‘4.3 Sense of loss of control over life’ - HRSQ-EP: ‘14. Hospital rules about issues such as bed time, time to eat, visiting hours, and time to take medication’ |
| 3. Having pain or discomfort from your treatment | - QCA: ‘Pain’ - HSRS: ‘19. Thinking you might have pain because of surgery or test procedures’; ‘19. Thinking you might have pain because of surgery or test procedures’; ‘28. Having medications cause you discomfort’; ‘40. Not getting relief from pain medications’; ‘42. Not getting pain medication when you need it’ - HRSQ-EP: ‘25. Being in pain due to the illness’ |
| 4. Staying in a noisy room | - QCA: ‘Noise’ - HSI: ‘7.2. Noise level on unit’ - HRSQ-EP: ‘16. Noise and traffic’ |
| 5. The staff not communicating well with you | - QCA: ‘Staff-patient communication’ - HSRS: ‘29. Having nurses or doctors talk too fast or use words you can’t understand’; ‘37. Not having your questions answered by the staff’ - HSI: ‘3.1.5. Doctors not speaking loudly or slowly enough’; ‘3.2.4. Nurses not speaking loudly or slowly enough’ - HRSQ-EP: ‘9. Use of unfamiliar words by doctor or the nurse’; ‘11. Communication of the staff with me’ |
| 6. The staff being too busy | - QCA: ‘Staff too busy’ - HSRS: ‘26. Having the staff be in too much of a hurry’ - HSI: ‘7.1. Insufficient nurses on unit to make patient feel safe’ - HRSQ-EP: ‘10. Doctor or the nurse not having enough time to respond to my needs’ |
| 7. The staff not being caring or friendly | - QCA: ‘Staff not caring or friendly’ |
| 8. Having to wait a lot | - QCA: ‘Waiting’ - HSI: ‘7.3. Problems during admission with waiting/other procedures’ |
| 9. The food being bad or not meeting your dietary requirements | - QCA: ‘Food unsuitable’ - HSRS: ‘21. Having to eat cold or tasteless food’ - HSI: ‘7.5. Inadequate facilities (food, recreation, room fixtures)’ - HRSQ-EP: ‘15. Hospital facilities such as room, bed, lighting, food, temperature and conditioning’ |
| 10. Feeling like you could not leave your bed or ward | - QCA: ‘Mobility restriction’; ‘Confined to bed or ward’ - HSRS: ‘10. Having to stay in bed or the same room all day’ - HRSQ-EP: ‘3. Mobility limitation due to the connected equipment’ |
| 11. Not knowing what was going to happen to you | - QCA: ‘Not knowing’ - HSRS: ‘26. Not knowing when to expect things will be done to you’; ‘41. Not knowing the results or reasons for your treatments’ - HSI: ‘4.1. Hospitalization and illness unexpected’; ‘5.2. Uncertainty of prognosis’ |
| 12. Feeling bored | - QCA: ‘Boredom’ - HSRS: ‘8. Not being able to get newspapers, radio, or TV when you want them’ - HSI: ‘7.5. Inadequate facilities (food, recreation, room fixtures)’ - HRSQ-EP: ‘1. Fatigue and impatience due to the long length of treatment’ |
| 13. The staff making a mistake that caused you harm | - QCA: ‘Staff made a mistake’ |
| 14. Worrying that your treatment/medication will have side effects | - QCA: ‘Adverse effects’ - HSRS: ‘32. Knowing you have to have an operation’ - HSI: ‘1.1. Adverse effects from a medical procedure’; ‘1.2. Adverse side-effects from medications’ - HRSQ-EP: ‘8. Not being confident in the care and treatment’; ‘22. Fear of death due to the risks of disease and treatment’ |
| 15. Fearing your health will get worse | - QCA: ‘Fear health would deteriorate’ - HSRS: ‘45. Thinking you might lose your hearing’; ‘46. Knowing you have a serious illness’; ‘47. Thinking you might lose a kidney or some other organ’; ‘48. Thinking you might have cancer’; ‘49. Thinking you might lose your sight’ - HSI: ‘8.1. Recurrent hospitalizations’; ‘8.3. Realization of how near the end is’ - HRSQ-EP: ‘8. Not being confident in the care and treatment’; ‘22. Fear of death due to the risks of disease and treatment’; ‘23. Worry about long lasting or persistent disability’; ‘24. Having another physical illness or disability besides current disease’ |
| 16. Feeling like the staff were not listening to you | - QCA: ‘Staff not listening’ - HSI: ‘3.1.2. Doctors don’t take complaints seriously’ |
| 17. The other patients being difficult | - QCA: ‘Disturbed by other patients’ - HSRS: ‘14. Having a roommate who is unfriendly’ |
| 18. Feeling lonely | - QCA: ‘Loneliness’ - HSRS: ‘15. Not having friends visit you’; ‘31. Not having family visit you’ - HSI: ‘4.4. Loneliness’ |
| 19. Missing loved ones | - QCA: ‘Missing loved ones’ - HSRS: ‘20. Worrying about your spouse being away from you’; ‘38. Missing your spouse’ - HRSQ-EP: ‘18. Limited contact with family and friends’ |
| 20. The staff being rude or unprofessional | - QCA: ‘Staff rude or unprofessional’ - HSI: ‘3.1.3. Lack of courtesy and respect from doctors while on rounds’; ‘3.2.3. Lack of courtesy and respect from nurses’ |
| 21. The staff not being responsive to the buzzer | - QCA: ‘Staff not responsive to buzzer or call’ - HSRS: ‘35. Not having your call light answered’ - HSI: ‘3.2.1. Nurses’ lack of responsiveness to patients needs’ - HRSQ-EP: ‘13. Staff not responding in a timely manner to my needs’ |
| 22. Having blood taken | - QCA: ‘Bloods taken’ - HSI: ‘1.3. Sleep disturbed by frequent tests, blood draws, medications’ - HRSQ-EP: ‘4. Diagnostic or therapeutic measures such as blood sampling or venipuncture or intravenous catheterizing’ |
| 23. Being disturbed by observations (e.g. blood pressure) | - QCA: ‘Disturbed by observations’ - HSRS: ‘6. Being awakened in the night by the nurse’ - HSI: ‘1.3. Sleep disturbed by frequent tests, blood draws, medications’ - HRSQ-EP: ‘4. Diagnostic or therapeutic measures such as blood sampling or venipuncture or intravenous catheterizing’ |
| 24. Having to rely on others | - QCA: ‘Relying on others’ - HSRS: ‘30. Feeling you are getting dependent on medications’ - HSI: ‘2.1. Difficulty with role transition to patient’; ‘4.2. Fear of dependency’ |
| 25. Not feeling safe | - QCA: ‘Feeling unsafe’ - HSI: ‘3.1.1. Lack of trust (doctor-patient)’; ‘3.2.2. Lack of trust (nurse-patient)’; ‘7.1. Insufficient nurses on unit to make patient feel safe’ |
| 26. Worrying about the wellbeing of other patients | - QCA: ‘Concern for other patients wellbeing’ - HSRS: ‘12. Having a roommate who is seriously ill or cannot talk with you’ |
| 27. The hospital not being organised | - QCA: ‘Hospital disorganised’ - HSI: ‘8.2. Poor transfer of information from PMD to hospital doctors’ |
| 28. Sharing a room with strangers | - QCA: ‘Sharing a room with other patients’ - HSRS: ‘1. Having strangers sleep in the same room with you’ |
| 29. The staff not communicating well with each other | - QCA: ‘Staff-staff communication’ - HSI: ‘8.2. Poor transfer of information from PMD to hospital doctors’ |
| 30. Not being allowed access to your usual medication | - QCA: ‘Access to own medication’ |
| 31. Hearing or seeing emergencies | - QCA: ‘Hearing or seeing emergencies’ |
| 32. Feeling homesick | - QCA: ‘Homesick’ - HSRS: ‘33. Being hospitalized far away from home’ - HSI: ‘6.1. Being away from home’ |
| 33. Not getting enough to drink | - QCA: ‘Hydration’ |
| 34. Being in an overcrowded ward | - QCA: ‘Overcrowding’ - HSRS: ‘9. Having a roommate who has too many visitors’ - HRSQ-EP: ‘16. Noise and traffic’ |
| 35. Overhearing the staff having conversations | - QCA: ‘Overhearing staff conversations’ - HSI: ‘3.1.6. Perceived disagreement among doctors on diagnosis or testing’ |
| 36. Medical procedure getting cancelled or delayed | - QCA: ‘Cancellation or delay’ - HSI: ‘1.4. Unexpected changes in scheduling or tests or procedures’ |
| 37. Not being involved in the treatment plan | - QCA: ‘Not involved in treatment plan’ |
| 38. Equipment or supplies lacking | - QCA: ‘Equipment lacking’ - HSI: ‘7.5. Inadequate facilities (food, recreation, room fixtures)’ - HRSQ-EP: ‘15. Hospital facilities such as room, bed, lighting, food, temperature and conditioning’ |
| 39. Having to deal with the symptoms of your illness (e.g. sickness) | - QCA: ‘Disturbed by symptoms’ - HSI: ‘8.4. Symptoms of illness’ |
| 40. Being in a room that was too hot or too cold | - QCA: ‘Temperature’ - HSRS: ‘16. Being in a room that is too cold or too hot’ - HSI: ‘7.5. Inadequate facilities (food, recreation, room fixtures)’ - HRSQ-EP: ‘15. Hospital facilities such as room, bed, lighting, food, temperature and conditioning’ |
| 41. Being in an unfamiliar place | - QCA: ‘Unfamiliar environment’ - HSRS: ‘3. Having to sleep in a strange bed’; ‘5. Having strange machines around’; ‘23. Being cared for by an unfamiliar doctor’ - HRSQ-EP: ‘6. Unfamiliarity with hospital’s environment’ |
| 42. Being in an unclean room | - QCA: ‘Lack of cleanliness’ - HSRS: ‘11. Being aware of unusual smells around you’ - HSI: ‘7.5. Inadequate facilities (food, recreation, room fixtures)’ - HRSQ-EP: ‘15. Hospital facilities such as room, bed, lighting, food, temperature and conditioning’ |
| 43. Worrying about loved ones | - QCA: ‘Worrying about loved ones’ - HSRS: ‘20. Worrying about your spouse being away from you’ - HSI: ‘6.2. Worry about the care of sick family members at home’; ‘6.3. Worry about who will care for family in future’ - HRSQ-EP: ‘20. Family disturbances’ |
| 44. Having to follow the hospital’s schedule | - QCA: ‘Having to follow hospital schedule’ - HSRS: ‘2. Having to eat at different times than you usually do’ - HSI: ‘Inflexibility of hospital routines’ - HRSQ-EP: ‘14. Hospital rules about issues such as bed time, time to eat, visiting hours, and time to take medication’; ‘17. Disruption of routine and normal habits and behaviors such as sleep and resting, activity, and diet’ |
| 45. Being in a room that was too bright or has no natural light | - QCA: ‘Lights’ - HSI: ‘7.5. Inadequate facilities (food, recreation, room fixtures)’ - HRSQ-EP: ‘15. Hospital facilities such as room, bed, lighting, food, temperature and conditioning’ |
| 46. Having tubes in your nose, mouth, or other body parts | - QCA: ‘Drips or tubes in body’ - HSRS: ‘39. Being fed through tubes’ - HRSQ-EP: ‘4. Diagnostic or therapeutic measures such as blood sampling or venipuncture or intravenous catheterizing’ |
| 47. Being reminded of loved ones who passed away while in hospital | - QCA: ‘Reminded of loved ones who passed away in hospital’ |
| 48. The staff not asking for consent before treating you | - QCA: ‘Staff did not ask for consent’ |
| 49. Not being able to do your usual activities | - QCA: ‘Cannot do normal activities’ - HSI: ‘2.2. Health problem limits activity’; ‘2.3. Physician imposed limits on diet, habits, or activity’ |
| 50. Fearing that you may pick up an illness from being in hospital | - QCA: ‘Fear of hospital acquired infections’ |
| 51. Feeling like you had no privacy | - QCA: ‘Lack of privacy’ - HRSQ-EP: ‘12. Staff not respecting my privacy’ |
| 52. Feeling like you were not being treated like a person | - QCA: ‘Dehumanisation’ |
| 53. Worrying about money | - QCA: ‘Financial stress’ - HSRS: ‘27. Thinking about losing income because of your illness’; ‘36. Not having enough insurance to pay for your hospitalization’ |
| 54. Feeling like your life was on hold or you were missing out | - QCA: ‘Life on hold’ - HSRS: ‘18. Being in the hospital during holidays or special family occasions’ |
| 55. Having poor Wi-Fi or phone signal | - QCA: ‘Poor/no Wi-Fi or phone signal’ - HSRS: ‘22. Not being able to call family or friends on the phone’ - HSI: ‘7.5. Inadequate facilities (food, recreation, room fixtures)’ - HRSQ-EP: ‘15. Hospital facilities such as room, bed, lighting, food, temperature and conditioning’ |
| 56. Not being sure of your diagnosis | - QCA: ‘Uncertain of diagnosis’ - HSRS: ‘43. Not knowing for sure what illness you have’; ‘44. Not being told what your diagnosis is’ - HSI: ‘5.1. Uncertainty of diagnosis’ - HRSQ-EP: ‘26. Low awareness about disease and treatment’ |
| 57. Worrying how you will cope once leaving hospital | - QCA: ‘Worrying post-hospital’ - HSI: ‘6.3. Worry about who will care for family in future’ |
| 58. Missing your usual small comforts (e.g. hot tea) | - QCA: ‘Missing small comforts’ |
| 59. Not being able to pray or do other religious activities | - QCA: ‘Not able to do religious activities’ - HRSQ-EP: ‘19. Difficulty doing religious obligations’ |
| 60. Feeling like the staff focused on other patients more than you | - QCA: ‘Staff attending to other patients more than you’ - HSI: ‘3.1.4. Doctors not spending enough time with patients’ |
| 61. Not knowing the hospital rules | - QCA: ‘Unfamiliar with hospital rules’ - HRSQ-EP: ‘5. Unfamiliarity with hospital’s rules’; ‘7. Being unaware of their own rights in the hospital’ |
| 62. Having to wear a hospital gown | - QCA: ‘Wearing a hospital gown’ - HSRS: ‘4. Having to wear a hospital gown’ |
| 63. Needing help going to the bathroom | - HSRS: ‘7. Having to be assisted with bathing’; ‘13. Having to be assisted with a bedpan’ - HSI: ‘8.5. Taking a bath’ - HSRQ-EP: ‘2. Requiring help for personal matters such as using catheter or else in bed’ |
| 64. Worrying that your appearance might change (e.g. scars) | - HSRS: ‘17. Thinking your appearance might be changed after your hospitalization’ - HRSQ-EP: ‘21. Worry about changes in body appearance due to illness and treatment’ |
| 65. Being transferred between wards or hospitals | - YQSR Group |
| 66. The hospital not meeting your individual needs (e.g. disability) | - YQSR Group |
| 67. Not being able to smoke, drink alcohol, or use other substances | - Research team |

*HRSQ-EP* Hospitalisation‐Related Stressors Questionnaire for Elderly Patients; *HSI* Hospital Stress Index; *HSRS* Hospital Stress Rating Scale; *QCA* Quantitative Content Analysis

**Appendix D – HSQ**

**Hospital Stress Questionnaire (HSQ)**

**What is the questionnaire about?**
This questionnaire aims to measure how much stress you experienced **during your hospital stay**. While answering the questions, consider 'stress' as feeling tense, worried, or wound up by a situation.

**Completing the questionnaire**
Please read each question carefully, keeping in mind your **most recent** stay in hospital and select one option for each question. **If you did not experience any of the events described in the questions**, please select N/A. This should take you around 5-10 minutes to complete. The best approach is to answer quickly.

| During your hospital stay, please rate how much stress you felt as a result of: | | | | | | | | | | | | |
| --- | --- | --- | --- | --- | --- | --- | --- | --- | --- | --- | --- | --- |
|  | Not at all stressful  (1) | | | | | | Extremely stressful  (10) | | | | |  |
| 1. Not sleeping well | 1 | 2 | 3 | 4 | 5 | 6 | | 7 | 8 | 9 | 10 | N/A |
| 2. Feeling helpless or not in control | 1 | 2 | 3 | 4 | 5 | 6 | | 7 | 8 | 9 | 10 | N/A |
| 3. Having pain or discomfort from your treatment | 1 | 2 | 3 | 4 | 5 | 6 | | 7 | 8 | 9 | 10 | N/A |
| 4. Staying in a noisy room | 1 | 2 | 3 | 4 | 5 | 6 | | 7 | 8 | 9 | 10 | N/A |
| 5. The staff not communicating well with you | 1 | 2 | 3 | 4 | 5 | 6 | | 7 | 8 | 9 | 10 | N/A |
| 6. The staff being too busy | 1 | 2 | 3 | 4 | 5 | 6 | | 7 | 8 | 9 | 10 | N/A |
| 7. The staff not being caring or friendly | 1 | 2 | 3 | 4 | 5 | 6 | | 7 | 8 | 9 | 10 | N/A |
| 8. Having to wait a lot | 1 | 2 | 3 | 4 | 5 | 6 | | 7 | 8 | 9 | 10 | N/A |
| 9. The food being bad or not meeting your dietary requirements | 1 | 2 | 3 | 4 | 5 | 6 | | 7 | 8 | 9 | 10 | N/A |
| 10. Feeling like you could not leave your bed or ward | 1 | 2 | 3 | 4 | 5 | 6 | | 7 | 8 | 9 | 10 | N/A |
| 11. Not knowing what was going to happen to you | 1 | 2 | 3 | 4 | 5 | 6 | | 7 | 8 | 9 | 10 | N/A |
| 12. Feeling bored | 1 | 2 | 3 | 4 | 5 | 6 | | 7 | 8 | 9 | 10 | N/A |
| 13. The staff making a mistake that caused you harm | 1 | 2 | 3 | 4 | 5 | 6 | | 7 | 8 | 9 | 10 | N/A |
| 14. Worrying that your treatment/medication will have side effects | 1 | 2 | 3 | 4 | 5 | 6 | | 7 | 8 | 9 | 10 | N/A |
| 15. Fearing your health will get worse | 1 | 2 | 3 | 4 | 5 | 6 | | 7 | 8 | 9 | 10 | N/A |
| 16. Feeling like the staff were not listening to you | 1 | 2 | 3 | 4 | 5 | 6 | | 7 | 8 | 9 | 10 | N/A |
| 17. The other patients being difficult | 1 | 2 | 3 | 4 | 5 | 6 | | 7 | 8 | 9 | 10 | N/A |
| 18. Feeling lonely | 1 | 2 | 3 | 4 | 5 | 6 | | 7 | 8 | 9 | 10 | N/A |
| 19. Missing loved ones | 1 | 2 | 3 | 4 | 5 | 6 | | 7 | 8 | 9 | 10 | N/A |
| 20. The staff being rude or unprofessional | 1 | 2 | 3 | 4 | 5 | 6 | | 7 | 8 | 9 | 10 | N/A |
| 21. The staff not being responsive to the buzzer | 1 | 2 | 3 | 4 | 5 | 6 | | 7 | 8 | 9 | 10 | N/A |
| 22. Having blood taken | 1 | 2 | 3 | 4 | 5 | 6 | | 7 | 8 | 9 | 10 | N/A |
| 23. Being disturbed by observations (e.g. blood pressure) | 1 | 2 | 3 | 4 | 5 | 6 | | 7 | 8 | 9 | 10 | N/A |
| 24. Having to rely on others | 1 | 2 | 3 | 4 | 5 | 6 | | 7 | 8 | 9 | 10 | N/A |
| 25. Not feeling safe | 1 | 2 | 3 | 4 | 5 | 6 | | 7 | 8 | 9 | 10 | N/A |
| 26. Worrying about the wellbeing of other patients | 1 | 2 | 3 | 4 | 5 | 6 | | 7 | 8 | 9 | 10 | N/A |
| 27. The hospital not being organised | 1 | 2 | 3 | 4 | 5 | 6 | | 7 | 8 | 9 | 10 | N/A |
| 28. Sharing a room with strangers | 1 | 2 | 3 | 4 | 5 | 6 | | 7 | 8 | 9 | 10 | N/A |
| 29. The staff not communicating well with each other | 1 | 2 | 3 | 4 | 5 | 6 | | 7 | 8 | 9 | 10 | N/A |
| 30. Not being allowed access to your usual medication | 1 | 2 | 3 | 4 | 5 | 6 | | 7 | 8 | 9 | 10 | N/A |
| 31. Hearing or seeing emergencies | 1 | 2 | 3 | 4 | 5 | 6 | | 7 | 8 | 9 | 10 | N/A |
| 32. Feeling homesick | 1 | 2 | 3 | 4 | 5 | 6 | | 7 | 8 | 9 | 10 | N/A |
| 33. Not getting enough to drink | 1 | 2 | 3 | 4 | 5 | 6 | | 7 | 8 | 9 | 10 | N/A |
| 34. Being in an overcrowded ward | 1 | 2 | 3 | 4 | 5 | 6 | | 7 | 8 | 9 | 10 | N/A |
| 35. Overhearing the staff having conversations | 1 | 2 | 3 | 4 | 5 | 6 | | 7 | 8 | 9 | 10 | N/A |
| 36. Medical procedure getting cancelled or delayed | 1 | 2 | 3 | 4 | 5 | 6 | | 7 | 8 | 9 | 10 | N/A |
| 37. Not being involved in the treatment plan | 1 | 2 | 3 | 4 | 5 | 6 | | 7 | 8 | 9 | 10 | N/A |
| 38. Equipment or supplies lacking | 1 | 2 | 3 | 4 | 5 | 6 | | 7 | 8 | 9 | 10 | N/A |
| 39. Having to deal with the symptoms of your illness (e.g. sickness) | 1 | 2 | 3 | 4 | 5 | 6 | | 7 | 8 | 9 | 10 | N/A |
| 40. Being in a room that was too hot or too cold | 1 | 2 | 3 | 4 | 5 | 6 | | 7 | 8 | 9 | 10 | N/A |
| 41. Being in an unfamiliar place | 1 | 2 | 3 | 4 | 5 | 6 | | 7 | 8 | 9 | 10 | N/A |
| 42. Being in an unclean room | 1 | 2 | 3 | 4 | 5 | 6 | | 7 | 8 | 9 | 10 | N/A |
| 43. Worrying about loved ones | 1 | 2 | 3 | 4 | 5 | 6 | | 7 | 8 | 9 | 10 | N/A |
| 44. Having to follow the hospital’s schedule | 1 | 2 | 3 | 4 | 5 | 6 | | 7 | 8 | 9 | 10 | N/A |
| 45. Being in a room that was too bright or has no natural light | 1 | 2 | 3 | 4 | 5 | 6 | | 7 | 8 | 9 | 10 | N/A |
| 46. Having tubes in your nose, mouth, or other body parts | 1 | 2 | 3 | 4 | 5 | 6 | | 7 | 8 | 9 | 10 | N/A |
| 47. Being reminded of loved ones who passed away while in hospital | 1 | 2 | 3 | 4 | 5 | 6 | | 7 | 8 | 9 | 10 | N/A |
| 48. The staff not asking for consent before treating you | 1 | 2 | 3 | 4 | 5 | 6 | | 7 | 8 | 9 | 10 | N/A |
| 49. Not being able to do your usual activities | 1 | 2 | 3 | 4 | 5 | 6 | | 7 | 8 | 9 | 10 | N/A |
| 50. Fearing that you may pick up an illness from being in hospital | 1 | 2 | 3 | 4 | 5 | 6 | | 7 | 8 | 9 | 10 | N/A |
| 51. Feeling like you had no privacy | 1 | 2 | 3 | 4 | 5 | 6 | | 7 | 8 | 9 | 10 | N/A |
| 52. Feeling like you were not being treated like a person | 1 | 2 | 3 | 4 | 5 | 6 | | 7 | 8 | 9 | 10 | N/A |
| 53. Worrying about money | 1 | 2 | 3 | 4 | 5 | 6 | | 7 | 8 | 9 | 10 | N/A |
| 54. Feeling like your life was on hold or you were missing out | 1 | 2 | 3 | 4 | 5 | 6 | | 7 | 8 | 9 | 10 | N/A |
| 55. Having poor Wi-Fi or phone signal | 1 | 2 | 3 | 4 | 5 | 6 | | 7 | 8 | 9 | 10 | N/A |
| 56. Not being sure of your diagnosis | 1 | 2 | 3 | 4 | 5 | 6 | | 7 | 8 | 9 | 10 | N/A |
| 57. Worrying how you will cope once leaving hospital | 1 | 2 | 3 | 4 | 5 | 6 | | 7 | 8 | 9 | 10 | N/A |
| 58. Missing your usual small comforts (e.g. hot tea) | 1 | 2 | 3 | 4 | 5 | 6 | | 7 | 8 | 9 | 10 | N/A |
| 59. Not being able to pray or do other religious activities | 1 | 2 | 3 | 4 | 5 | 6 | | 7 | 8 | 9 | 10 | N/A |
| 60. Feeling like the staff focused on other patients more than you | 1 | 2 | 3 | 4 | 5 | 6 | | 7 | 8 | 9 | 10 | N/A |
| 61. Not knowing the hospital rules | 1 | 2 | 3 | 4 | 5 | 6 | | 7 | 8 | 9 | 10 | N/A |
| 62. Having to wear a hospital gown | 1 | 2 | 3 | 4 | 5 | 6 | | 7 | 8 | 9 | 10 | N/A |
| 63. Needing help going to the bathroom | 1 | 2 | 3 | 4 | 5 | 6 | | 7 | 8 | 9 | 10 | N/A |
| 64. Worrying that your appearance might change (e.g. scars) | 1 | 2 | 3 | 4 | 5 | 6 | | 7 | 8 | 9 | 10 | N/A |
| 65. Being transferred between wards or hospitals | 1 | 2 | 3 | 4 | 5 | 6 | | 7 | 8 | 9 | 10 | N/A |
| 66. The hospital not meeting your individual needs (e.g. disability) | 1 | 2 | 3 | 4 | 5 | 6 | | 7 | 8 | 9 | 10 | N/A |
| 67. Not being able to smoke, drink alcohol, or use other substances | 1 | 2 | 3 | 4 | 5 | 6 | | 7 | 8 | 9 | 10 | N/A |
| Other (write in) | 1 | 2 | 3 | 4 | 5 | 6 | | 7 | 8 | 9 | 10 | N/A |

Overall, how stressed did you feel during your hospital stay?

| No stress (1) | | | | | Worst possible stress (10) | | | | |
| --- | --- | --- | --- | --- | --- | --- | --- | --- | --- |
| 1 | 2 | 3 | 4 | 5 | 6 | 7 | 8 | 9 | 10 |

If you have any additional comments, please write them here:

________________________________________________________________________________________________________________________________________________________________________________________________
